# The legacy effect of hyperglycemia and early use of SGLT-2 inhibitors: a cohort study with newly-diagnosed people with type 2 diabetes

**DOI:** 10.1016/j.lanepe.2023.100666

**Published:** 2023-06-12

**Authors:** Antonio Ceriello, Giuseppe Lucisano, Francesco Prattichizzo, Rosalba La Grotta, Chiara Frigé, Salvatore De Cosmo, Paolo Di Bartolo, Graziano Di Cianni, Paola Fioretto, Carlo Bruno Giorda, Roberto Pontremoli, Giuseppina Russo, Francesca Viazzi, Antonio Nicolucci, AMD Annals study group

**Affiliations:** a IRCCS MultiMedica, Milan, Italy; b CORESEARCH - Center for Outcomes Research and Clinical Epidemiology, Pescara, Italy; c Department of Medical Sciences, Scientific Institute “Casa Sollievo della Sofferenza”, San Giovanni Rotondo, FG, Italy; d Ravenna Diabetes Center, Department of Specialist Medicine, Romagna Local Health Authority, Italy; e Diabetes Unit Livorno Hospital, Italy; f Department of Medicine, University of Padua, Unit of Medical Clinic 3, Hospital of Padua, Padua, Italy; g Diabetes and Metabolism Unit, ASL Turin 5, Chieri, TO, Italy; h IRCCS Ospedale Policlinico San Martino; Dipartimento di Medicina Interna, Università degli studi di Genova, Genoa, Italy; i Department of Clinical and Experimental Medicine, University of Messina, Messina, Italy; j AMD Foundation, Roma, Italy

**Keywords:** AMD Annals initiative, Type 2 diabetes, Metabolic memory, Legacy effect, Cardiovascular diseases, Sodium-glucose cotransporter 2 inhibitors

## Abstract

**Background:**

A delay in reaching HbA1c targets in patients with newly-diagnosed type 2 diabetes (T2D) is associated with an increased long-term risk of developing cardiovascular diseases (CVD), a phenomenon referred to as legacy effect. Whether an early introduction of glucose-lowering drugs with proven benefit on CVD can attenuate this phenomenon is unknown.

**Methods:**

Using data derived from a large Italian clinical registry, *i.e*. the AMD Annals, we identified 251,339 subjects with newly-diagnosed T2D and without CVD at baseline. Through Cox regressions adjusted for multiple risk factors, we examined the association between having a mean HbA1c between 7.1 and 8% or >8%, compared with ≤7%, for various periods of early exposure (0–1, 0–2, 0–3 years) and the development of later (mean subsequent follow-up 4.6 ± 2.9 years) CVD, evaluated as a composite of myocardial infarction, stroke, coronary or peripheral revascularization, and coronary or peripheral bypass. We performed this analysis in the overall cohort and then splitting the population in two groups of patients: those that introduced sodium-glucose transport protein 2 inhibitors (SGLT-2i) during the exposure phase and those not treated with these drugs.

**Findings:**

Considering the whole cohort, subjects with both a mean HbA1c between 7.1 and 8% and >8%, compared with patients attaining a mean HbA1c ≤ 7%, showed an increased risk of developing the outcome in all the three early exposure periods assessed, with the highest risk observed in patients with mean HbA1c > 8% in the 3 years exposure period (hazard ratio [HR]1.33; 95% confidence interval [CI] 1.063–1.365). The introduction of SGLT-2i during the exposure periods of 0–1 and 0–2 years eliminated the association between poor glycemic control and the outcome (p for interaction 0.006 and 0.003, respectively, vs. patients with the same degree of glycemic control but not treated with these drugs).

**Interpretation:**

Among patients with newly diagnosed T2D and free of CVD at baseline, a poor glycemic control in the first three years after diagnosis is associated with an increased subsequent risk of CVD. This association is no longer evident when SGLT-2i are introduced in the first two years, suggesting that these drugs attenuate the phenomenon of legacy effect. An early treatment with these drugs might thus promote a long-lasting benefit in patients not attaining proper glycemic control after T2D diagnosis.

**Funding:**

This work was supported, in part, by the Italian 10.13039/100009647Ministry of Health (Ricerca Corrente) to IRCCS MultiMedica.

**Research in context**

**Evidence before this study**

Data from historic clinical trials and the subsequent observational follow-ups such as the UKPDS and the DCCT/EDIC, as well as large observational studies such as the Diabetes & Aging study, suggest that a poor glycemic control in patients with early-stage diabetes increases the long-term risk of macrovascular complications, a phenomenon known as the legacy effect. However, such studies were conducted when glucose-lowering drugs with proven cardiovascular benefit, *e.g*., the SGLT-2i, were not available.

**Added value of this study**

With this study we substantiate the evidence that poor glycemic control after the diagnosis of type 2 diabetes is associated with an increased cardiovascular risk during the subsequent follow-up. However, we show also that the introduction of an SGLT-2i during the first two years after diabetes diagnosis eliminated the association between poor glycemic control and the later development of cardiovascular events, measured as a composite of myocardial infarction, stroke, coronary or peripheral revascularization, and coronary or peripheral bypass.

**Implications of all the available evidence**

While the results of this study need confirmation in independent and prospective cohorts, they might suggest that SGLT-2i could attenuate the deleterious long-term damage promoted by poor glycemic control in the first years after diabetes diagnosis. Thus, while reaching the HbA1c target as soon as possible remains the main therapeutic goal in early diabetes management, the introduction of SGLT-2i might be considered an option for those patients unable to attain rapidly the recommended HbA1c target.

## Introduction

Patients with type 2 diabetes (T2D) have an increased risk of cardiovascular diseases (CVD) and poor glycemic control is a key risk factor in this population.^1^^,^^2^ An early and intensive glycemic control has been associated with a long-term benefit on the development of CVD, a phenomenon referred to as legacy effect.^3^ Indeed, findings from the follow-up of the UKPDS trial suggested that patients with a recent T2D diagnosis benefit from an intensive glycemic control even after the intensive therapy is discontinued.^4^ Albeit selected, subsequent studies enrolling patients with more advanced stages of T2D did not confirm these results.^5^, ^6^, ^7^ On the contrary, two large observational studies and additional, recent, follow-up data of the same UKPDS cohort provided consistent evidence that newly diagnosed T2D patients with various degrees of poor glycemic control in the years following diagnosis have an increased risk of late CVD and death, supporting the existence of a long-lasting damage promoted by hyperglycemia.^8^, ^9^, ^10^ However, all the studies exploring the phenomenon of legacy effect were conducted when glucose-lowering drugs with proven cardiovascular benefit, *e.g*., sodium-glucose transport protein 2 inhibitors (SGLT-2i), were not available. Thus, whether an early introduction of these drugs blunt the deleterious consequence of poor glycemic control after T2D diagnosis is unknown.

SGLT-2i have a demonstrated ability to lower the incidence of cardiovascular and other outcomes. In particular, multiple trials have demonstrated that SGLT-2i reduce the incidence of cardiovascular mortality, heart failure, and kidney-related events including the development of albuminuria in patients with T2D,^11^, ^12^, ^13^, ^14^ while large cohort studies suggest also a benefit on atherosclerotic endpoints in this population.^15^ Several mediation analyses indicate that the effect of these drugs on multiple, canonical risk factors, including attained HbA1c levels, unlikely explain the observed effect on hard outcomes,^16^^,^^17^ thus suggesting that the benefit might derive from peculiar mechanisms attributable to this class. Among other frameworks, it was hypothesized that these drugs are able to antagonize the major pathological imbalances of T2D, thus potentially changing the trajectory of the disease.^18^, ^19^, ^20^ As a corollary of this postulate, an early introduction of SGLT-2i should thus promote a long-term beneficial effect on the vasculature independently of the attained HbA1c targets.

To explore this hypothesis, we took advantage of a large Italian clinical registry of people with T2D, the AMD Annals Initiative, to extrapolate data from newly-diagnosed patients and free of CVD at baseline, in order to examine the association between attained HbA1c targets in the years following T2D diagnosis and later CVD in the whole cohort and then in two populations of patients: those introducing early an SGLT-2i and patients not treated with these drugs during the same period.

## Methods

### Study design and population

Data derived from the registry of the Italian Association of Clinical Diabetologists [Associazione Medici Diabetologi (AMD)] Annals initiative, which was established in 2004 to monitor quality of diabetes care in Italy.^21^ The database includes information on all patients with T2DM receiving care at 230 diabetes clinics in Italy from January 1st 2004 to December 31st 2021. All diabetes clinics adhering to AMD Annals initiative, a third of those existing throughout the country, used a common electronic clinical record system for the everyday management of outpatients and a software was specifically developed to extract information from these clinical databases. Anonymized data from all participating clinics were collected and centrally analyzed. Available data included demographic, clinical, and biochemical information, including values of glycated haemoglobin (HbA1c), blood pressure, total-cholesterol, low-density lipoprotein cholesterol (LDL-C) or high-density lipoprotein cholesterol (HDL-C), and triglycerides. The use of specific classes of drugs (glucose lowering, lipid lowering and antihypertensive agents), based on ATC codes, was available. Information on the presence of diabetic complications was based on ICD-9 CM codes.

**Fig. 1:**
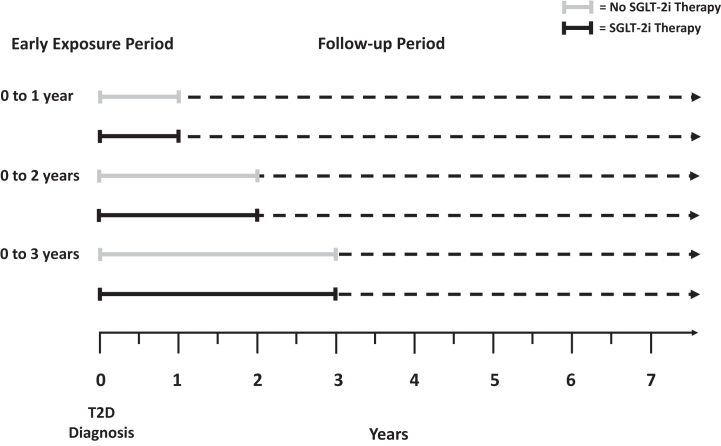
**Schematic representation of the experimental design**.

The experimental design is summarized in Fig. 1. To explore the effects of various periods of early glycemic exposure, we defined three definition of early exposure periods (0–1, 0–2 and 0–3 years). The mean HbA1c value was calculated for each early exposure period by using all HbA1c results except the value at diagnosis. The value at diagnosis was excluded because it reflects control before treatment was initiated, and the glycemic legacy effect has been demonstrated only in populations receiving diabetes treatment. To assess also the effect of various degrees of glycemic control, the mean HbA1c value for each of the three early exposure periods was categorized into either HbA1c ≤ 7.0% (≤53 mmol/mol), >7.0%–≤8.0% (>53–≤64 mmol/mol), >8.0% (>64 mmol/mol). The exposure period starts at the diagnosis date and end after first, second or third year from diagnosis. The follow-up period starts after first, second or third year from diagnosis (baseline/t0) and was ended after the first occurrence of the outcome of interest or censored at last visit. The outcome of interest was the composite of myocardial infarction, stroke, coronary or peripheral revascularization, and coronary or peripheral bypass. Patients with prevalent CVD at baseline were excluded. The risk factors used to adjust the analysis derived from the last observed values for each of the three early exposure periods (baseline/t0). When a variable was not available at baseline, it was carried backward in the previous years. In case of missing data relative to covariates, a category of missing data was added for each covariate in the multivariate analysis. This design was adopted for both the analysis of the whole cohort and for the analyses stratified according to the use of SGLT-2i during the exposure phase vs. non-user (Fig. 1). Patients could have introduced the drug at any moment during the exposure phase considered, thus the subgroup using SGLT-2i in the 0–3 years exposure period include also those subjects introducing the drugs in the 0–1 year and 0–2 years’ exposure periods, while those of the 0–2 years exposure phase include also patients introducing the drugs in the first year after diagnosis. This design was selected to maximize the chances of observing an effect of the drug against the legacy effect.

### Statistical analysis

We summarized data for patient characteristics using means and standard deviations (SDs) for continuous variables and counts and percentages for categorical variables stratified by the three classes of HbA1c mean in early exposure period. The characteristics were compared by the t-test and χ2 test respectively for continuous and categorical variables.

The Cox proportional hazards models were used to examine associations between glycemic control and the risk of CVD. The Cox model were adjusted for potentially confounding variables: sex (male vs. female), age (by 5 years), total-cholesterol (by 10 mmol/l), HDL-cholesterol (by 10 mmol/l) and LDL-cholesterol (by 10 mmol/l), triglycerides (by 10 mmol/l), BMI, systolic blood pressure (by 5 mmHg), smoking status (Yes vs. No), eGFR (ml/min/1.73m^2^ <60 vs. ≥60), microalbuminuria (Yes vs. No), the use of different classes of glucose-lowering drugs (Yes vs. No for each one), statin (Yes vs. No), antihypertensive medication (Yes vs. No), HbA1c, and the number of HbA1c measurements during the exposure period. A backward selection was introduced in the Cox models in order to exclude the confounders without a significant association with the outcome. The descriptive and multivariate analysis were performed three cohorts. Then, the population was stratified for SGLT2 use during the exposure phase or no-use and the Cox models were run separately for these populations. p-values for treatment by subgroup interaction were obtained from tests of homogeneity of treatment group differences among subgroups. For each model, patient follow-up was censored after the first occurrence of the outcome of interest or last visit. A two-sided p < 0.05 was considered statistically significant for all analyses. Analyses were performed using SAS 9.4 statistical software (SAS Institute, Cary, NC).

### Role of the funding source

The funders had no role in study design, data collection, data analysis, interpretation or writing of the report.

## Results

### Early poor glycemic control is associated with later risk of cardiovascular diseases

**Fig. 2:**
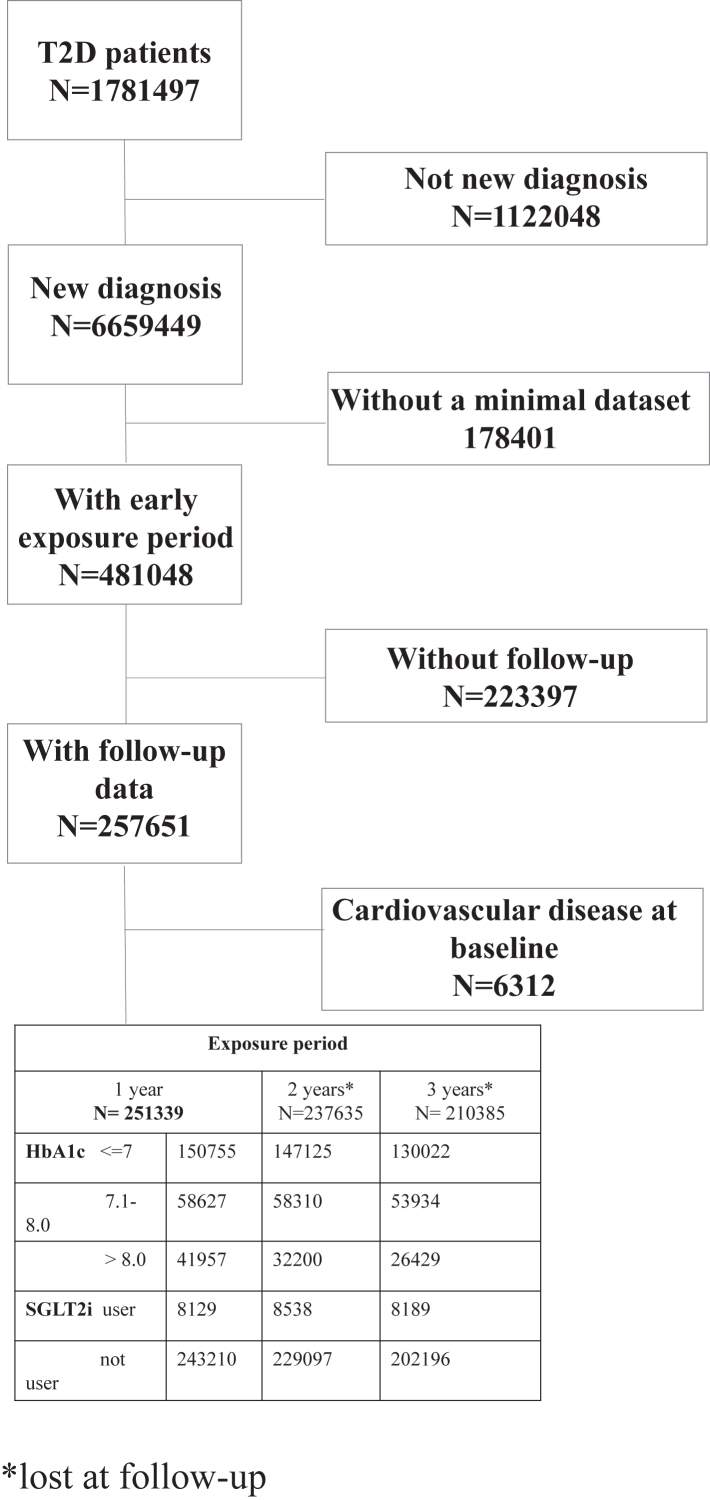
**Flow-diagram showing included and excluded patients**.

From the AMD Annals database, 251,339 patients with newly diagnosed T2D and free of CVD at baseline were identified and included in the study (Fig. 2). Clinical characteristics of the groups categorized according to the degree of glycemic control attained in the three different exposure periods are summarized in Supplementary Table S1A–C. The groups showed significant differences for almost all the cardiovascular risk factor assessed. Thus, all these variables were added as covariates to adjust the subsequent analyses.

**Fig. 3:**
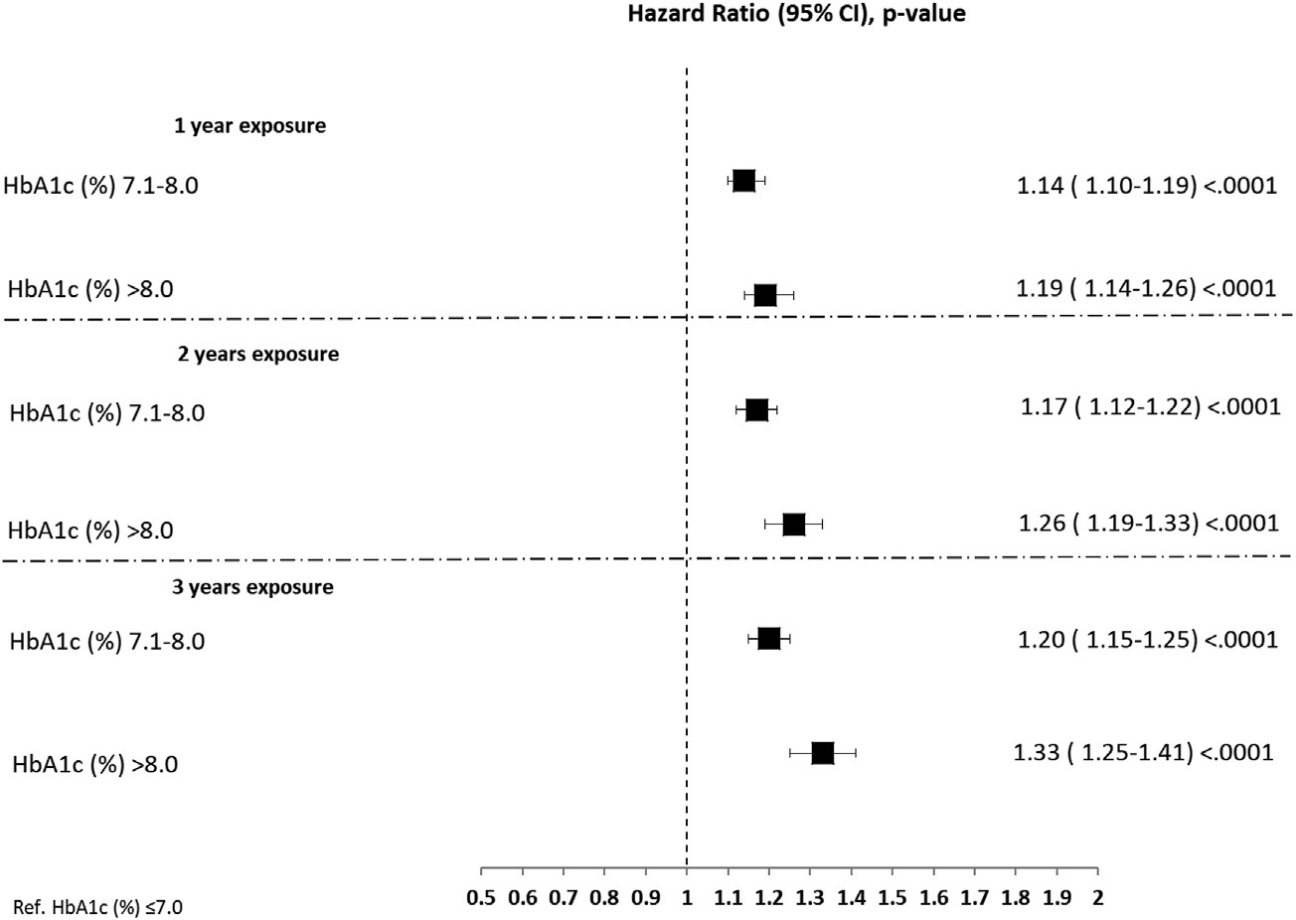
**Poor, early glycemic control and the subsequent risk of cardiovascular diseases.** Pseudo-forest plot showing the adjusted hazard ratios (HR) with the relative 95% confidence interval (CI) and the p value, derived from the Cox regression analyses exploring the associations between glycemic control and the risk of the CVD at follow-up in the whole cohort according to the degree of glycemic control in the three exposure periods assessed. HbA1c ≤ 7% is the reference.

Cox regression analysis, adjusted for sex, age, total-cholesterol, HDL-cholesterol, LDL-cholesterol, triglycerides, BMI, systolic blood pressure, smoking status, eGFR, microalbuminuria, the use of different classes of glucose-lowering drugs, statin, antihypertensive medication, HbA1c, and the number of HbA1c measurements, showed that, compared with patients with a mean HbA1c ≤ 7%, those above this range had in increased risk of CVD at follow-up for all the three early exposure periods considered and for both strata of poor glycemic control considered (Fig. 3). In detail, compared with HbA1c ≤ 7%, patients with mean HbA1c > 7 and <8% had a hazard ratio [HR] of 1.14, 95% confidence interval [CI] 1.10–1.19 for the 0–1-year exposure period, a HR of 1.17; 95%, CI 1.12–1.22 for the 0–2 years exposure period, and a HR 1.20, 95% CI 1.15–1.25 for the 0–3 years exposure period (p < 0.0001 for all). Similar data were obtained for patients with mean HbA1c > 8% (HR 1.19; 95%, CI 1.14–1.26 for the 0–1-year exposure period, HR 1.26; 95%, CI 1.19–1.33 for the 0–2 years exposure period, and HR 1.33, 95% CI 1.25–1.41 for the 0–3 years exposure period, p < 0.0001 for all) (Fig. 3). There was a significant trend toward an increasing risk of CVD with progressively higher levels of mean HbA1c in each of the exposure periods assessed (p for trend <0.0001 for all).

### Introduction of SGLT-2i in the first two years after diagnosis ameliorates the legacy effect

**Fig. 4:**
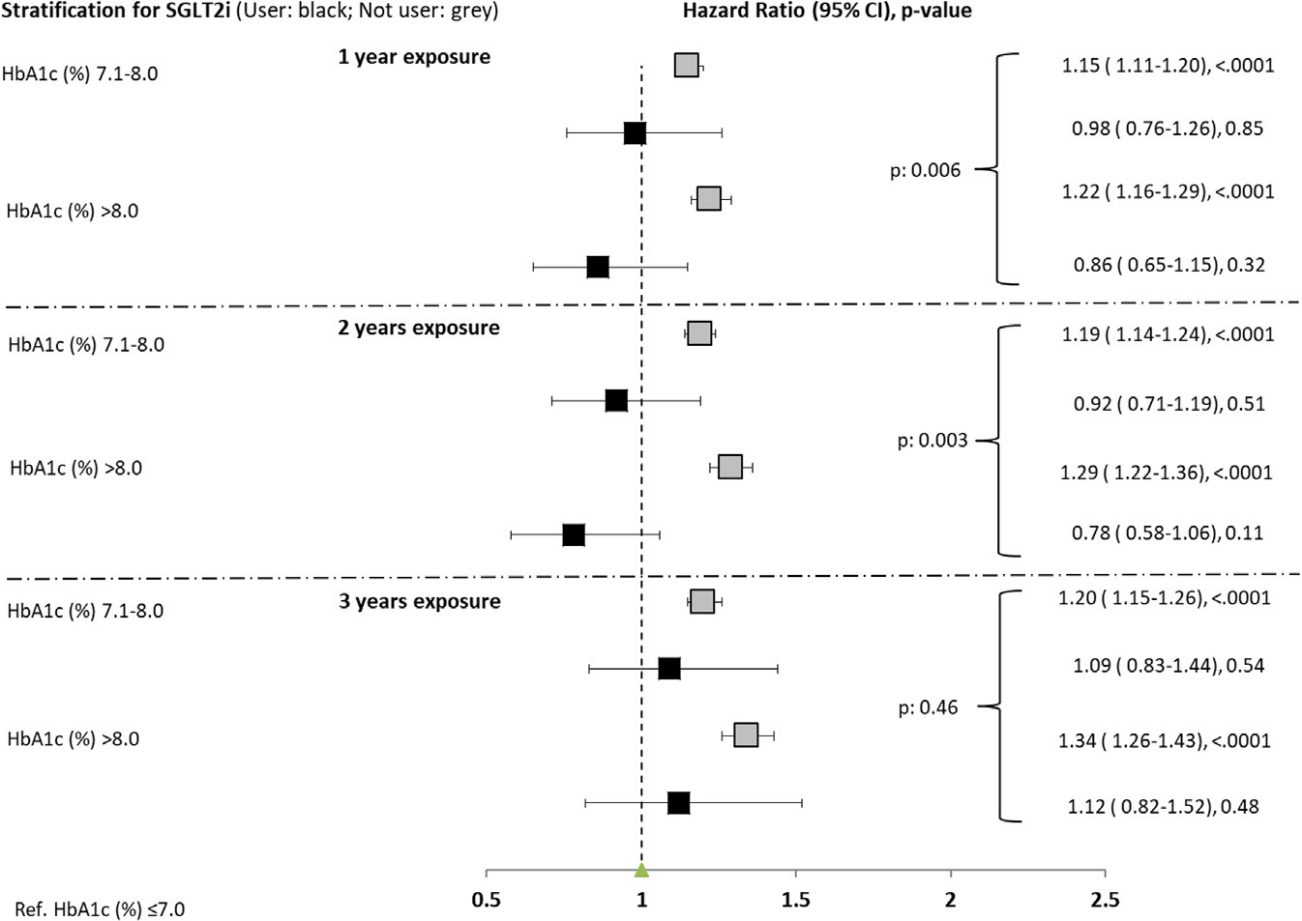
**Early introduction of SGLT-2i attenuate metabolic memory.** Pseudo-forest plot showing the adjusted hazard ratios (HR) with the relative 95% confidence interval (CI) and the p value, derived from the Cox regression analyses exploring the associations between glycemic control and the risk of the CVD at follow-up in patients stratified according to use of SGLT-2i during the exposure phase or not users, in the three exposure periods assessed. HbA1c ≤ 7% is the reference.

We then split the cohort in two populations: those introducing SGLT-2i during any time of the exposure phase considered and non-users. Clinical characteristics of the groups in the three exposure periods are presented in Table 1. All the significantly different risk factors were used to adjust the Cox models. As shown in Fig. 4, the introduction of SGLT-2i in the 0–1 year and 0–2 years exposure phases blunted the association between poor glycemic control and later CVD (HR 0.98; 95%, CI 0.76–1.26 in users vs. HR 1.15; 95%, CI 1.11–1.20 in non-users for the 7% < HbA1c mean ≤8% strata and HR 0.86; 95%, CI 0.65–1.15 in users vs. HR 1.22; 95%, CI 1.16–1.29 in non-users for the mean HbA1c > 8% strata in the 0–1-year exposure period, p for interaction 0.006; HR 0.92; 95%, CI 0.71–1.19 in users vs. HR 1.19; 95%, CI 1.14–1.24 in non-users for the 7% < HbA1c mean ≤8% strata and HR 0.78; 95%, CI 0.58–1.06 in users vs. HR 1.29; 95%, CI 1.22–1.36 in non-users for the mean HbA1c > 8% strata in the 0–2 years exposure period, p for interaction 0.003). This phenomenon was not observed when SGLT-2i were introduced in the 0–3 years exposure phase (p for interaction = 0.46).

**Table 1:** Characteristics of the study population by SGLT2i use in three exposure periods.

Variable	1 year exposure			2 years exposure			3 years exposure		
	Not user	User	p-value	Not user	User	p-value	Not user	User	p-value
No. of patients	243,210	8129		229,097	8538		202,196	8189	
Age at baseline (year)	63.8 ± 12.5	58.9 ± 10.2	<0.0001	64.5 ± 12.3	58.9 ± 10.1	<0.0001	65.1 ± 12.2	58.9 ± 10.1	<0.0001
Gender (% males)	137,996 (56.7)	5170 (63.6)	<0.0001	129,167 (56.4)	5335 (62.5)	<0.0001	113,652 (56.2)	5061 (61.8)	<0.0001
Microalbuminuria	47,728 (31.3)	2117 (35.1)	<0.0001	58,231 (35.4)	2867 (40.4)	<0.0001	61,181 (39.2)	3190 (45.0)	<0.0001
Antihypertensive medication	115,342 (47.4)	4500 (55.4)	<0.0001	118,426 (51.7)	5020 (58.8)	<0.0001	110,785 (54.8)	4917 (60.0)	<0.0001
BMI	29.8 ± 5.6	31.4 ± 6.0	<0.0001	29.8 ± 5.6	31.8 ± 6.1	<0.0001	29.9 ± 5.5	32.2 ± 6.2	<0.0001
Total cholesterol (mmol/l)	187.7 ± 40.7	181.5 ± 42.4	<0.0001	184.7 ± 39.4	179.5 ± 40.9	<0.0001	182.6 ± 38.9	177.9 ± 40.2	<0.0001
eGFR (ml/min/1.73m^2^) <60	37,587 (18.1)	757 (9.8)	<0.0001	41,170 (20.2)	932 (11.2)	<0.0001	40,672 (22.1)	990 (12.3)	<0.0001
Follow-up (years)	4.7 ± 2.9	2.1 ± 1.3	<0.0001	4.6 ± 2.7	2.0 ± 1.2	<0.0001	4.4 ± 2.4	2.0 ± 1.2	<0.0001
HbA1c at baseline (%)	6.7 ± 1.1	7.0 ± 1.3	<0.0001	6.8 ± 1.1	7.1 ± 1.2	<0.0001	6.8 ± 1.1	7.3 ± 1.2	<0.0001
HbA1c at diagnosis (%) 3.0–6.9	45,985 (18.9)	552 (6.8)	<0.0001	46,679 (20.4)	632 (7.4)	<0.0001	42,287 (20.9)	697 (8.5)	<0.0001
HbA1c at diagnosis (%) 7.0–8.0	34,370 (14.1)	894 (11.0)		32,418 (14.2)	976 (11.4)		28,219 (14.0)	937 (11.4)	
HbA1c at diagnosis (%) 8.1–9.0	15,349 (6.3)	701 (8.6)		13,798 (6.0)	696 (8.2)		11,905 (5.9)	651 (7.9)	
HbA1c at diagnosis (%) >9.0	38,624 (15.9)	2785 (34.3)		33,398 (14.6)	2706 (31.7)		28,032 (13.9)	2408 (29.4)	
HbA1c at diagnosis (%) NA	8.3 ± 2.3	9.7 ± 2.3	<0.0001	8.2 ± 2.2	9.6 ± 2.4	<0.0001	8.1 ± 2.2	9.5 ± 2.4	<0.0001
HbA1c at diagnosis (%)	49.1 ± 13.1	47.7 ± 12.3	<0.0001	49.5 ± 13.3	47.6 ± 12.5	<0.0001	49.6 ± 13.4	47.2 ± 12.3	<0.0001
HDL cholesterol (mmol/l)	110.9 ± 35.0	104.6 ± 35.9	<0.0001	107.6 ± 34.0	101.9 ± 34.8	<0.0001	105.5 ± 33.6	100.1 ± 33.8	<0.0001
LDL cholesterol (mmol/l)	147,507 (60.7)	3248 (40.0)	<0.0001	143,846 (62.8)	3279 (38.4)	<0.0001	127,113 (62.9)	2909 (35.5)	<0.0001
HbA1c mean in exposure period ≤7.0	56,211 (23.1)	2416 (29.7)		55,277 (24.1)	3033 (35.5)		50,759 (25.1)	3175 (38.8)	
HbA1c mean in exposure period 7.1–8.0	39,492 (16.2)	2465 (30.3)		29,974 (13.1)	2226 (26.1)		24,324 (12.0)	2105 (25.7)	
HbA1c mean in exposure period >8.0	78.8 ± 9.9	79.8 ± 10.2	<0.0001	78.8 ± 9.9	79.8 ± 9.8	<0.0001	78.7 ± 9.7	79.8 ± 9.9	<0.0001
Diastolic blood pressure (mmHg)	133.9 ± 18.1	133.5 ± 18.2	0.005	134.2 ± 17.9	133.1 ± 17.3	<0.0001	134.4 ± 17.8	133.6 ± 17.6	<0.0001
Systolic blood pressure (mmHg)	18,874 (7.8)	376 (4.6)	<0.0001	22,818 (10.0)	740 (8.7)	<0.0001	23,928 (11.8)	1098 (13.4)	<0.0001
Smoking	30,907 (20.1)	1367 (24.8)	<0.0001	29,680 (19.8)	1532 (25.3)	<0.0001	26,554 (19.6)	1481 (24.8)	<0.0001
Statins	82,775 (34.0)	3893 (47.9)	<0.0001	92,140 (40.2)	4683 (54.8)	<0.0001	90,106 (44.6)	4797 (58.6)	<0.0001
Composite CV Outcome	13,485 (5.5)	337 (4.1)	<0.0001	12,596 (5.5)	302 (3.5)	<0.0001	10,864 (5.4)	282 (3.4)	<0.0001
Exposure time (year)	0.7 ± 0.3	0.8 ± 0.2	<0.0001	1.6 ± 0.5	1.7 ± 0.3	<0.0001	2.4 ± 0.7	2.7 ± 0.4	<0.0001
Triglycerides (mmol/l)	143.4 ± 89.0	154.7 ± 102.5	<0.0001	142.4 ± 86.9	159.3 ± 104.1	<0.0001	142.3 ± 85.8	163.6 ± 106.3	<0.0001
DPP4i	7843 (3.2)	65 (0.8)	<0.0001	8878 (3.9)	109 (1.3)	<0.0001	9093 (4.5)	155 (1.9)	<0.0001
Glinides	5008 (2.1)	140 (1.7)	0.0349	6121 (2.7)	238 (2.8)	0.5152	6639 (3.3)	358 (4.4)	<0.0001
GLP1-RAs	5113 (2.1)	328 (4.0)	<0.0001	6023 (2.6)	669 (7.8)	<0.0001	6337 (3.1)	980 (12.0)	<0.0001
Acarbose	3643 (1.5)	58 (0.7)	<0.0001	4404 (1.9)	94 (1.1)	<0.0001	4661 (2.3)	154 (1.9)	0.0118
Insulin	42,306 (17.4)	2641 (32.5)	<0.0001	39,855 (17.4)	2966 (34.7)	<0.0001	36,113 (17.9)	3005 (36.7)	<0.0001
Metformin	140,715 (57.9)	7409 (91.1)	<0.0001	140,670 (61.4)	7934 (92.9)	<0.0001	129,717 (64.2)	7701 (94.0)	<0.0001
Sulphonylureas	23,452 (9.6)	497 (6.1)	<0.0001	26,166 (11.4)	861 (10.1)	0.0001	26,560 (13.1)	1130 (13.8)	0.0818

To corroborate the latter findings, we extended the exposure phase adding a 0–4 years exposure period. Patients’ characteristics are shown in Supplementary Table S1D. Even in this case, we did not observe a significant interaction between patients introducing or not introducing SGLT-2i during the exposure phase (Supplementary Table S2, p for interaction = 0.14), possibly suggesting that the time window to attenuate the legacy effect might be limited.

Finally, we checked the persistence rate of SGLT-2i prescription during the follow-up. Data are shown in Supplementary Table S3 and suggest that discontinuation rate ranged from 12.6% to 13.7% in the four exposure periods considered.

## Discussion

The burden of evidence showing the ability of SGLT-2i to halt the progression of CVD and renal failure in T2D, coupled by mechanistic data highlighting their effects on major pathophysiological abnormalities of T2D, suggest them as potential disease-modifying drugs.^18^, ^19^, ^20^ If true, the benefit of an early introduction of these drugs should be long-lasting, independently of the degree of glycemic control. Legacy effect is a well-recognized phenomenon clearly emerged in cohort studies and in selected clinical trials, which envisages that poor glycemic control after T2D diagnosis promote an enduring damage on the vasculature.^3^ Here we show for the first time that an early introduction of SGLT-2i is able to eliminate the association between poor glycemic control in the first two years after T2D diagnosis and the later development of CVD, independently of the glycemic control attained. If confirmed, these data might sustain the argument that these drugs actually work as disease-modifying drugs, an observation with obvious clinical implications.^18^^,^^22^^,^^23^

The results relative to the overall cohort are similar to those observed previously.^8^, ^9^, ^10^ For instance, in the Diabetes and Aging study, patients with HbA1c levels ≥6.5% for the 0-to-1-year early exposure period had a higher risk for late macrovascular events, with this risk being progressively higher for longer periods of exposure to very poor glycemic control, *i.e.* HbA1c levels ≥9.0%.^9^ Our results are also compatible with a framework where the damage promoted by poor glycemic control after T2D diagnosis has a progressive nature in terms of both years of exposure and HbA1c levels, albeit further studies are required to clarify these issues. On the other hand, the introduction of SGLT-2i in the first two years after diagnosis, but not in the 0–3 years and 0–4 years periods, blunted this association, possibly suggesting that the time window to change the pathological trajectory induced by poor glycemic control might be limited. While these results need to be substantiated and expanded, our observation might sustain the argument that the beneficial effects of SGLT-2i on CVD might be mediated by early direct effects of these drugs on T2D pathogenetic mechanisms, with long-term consequences on the vasculature.

SGLT-2i are the only glucose lowering drugs not needing the action of insulin to induce glucose clearance, promoting also a net elimination of calories and fluids. This action fosters a metabolic shift at the systemic level, lowering insulinemia and increasing the glucagon/insulin ratio, finally promoting ketones and fatty acids utilization as alternative substrates.^24^^,^^25^ This metabolic, hormonal, and hemodynamic reshaping is accompanied by the modulation of a number of major pathways and phenomena mainly underlying the typical pathological imbalances of T2D or the most relevant risk factors for CVD, such as hypertension, obesity, liver dysfunction, kidney disease, beta-cell dysfunction, low-grade inflammation, endothelial dysfunction, and insulin resistance in multiple tissues.^19^^,^^20^^,^^26^, ^27^, ^28^ In addition, SGLT-2i have molecular data supporting their ability to counteract the activation of a large range of detrimental mechanisms held to underlie the legacy effect, *e.g*., long-lasting oxidative stress, non-enzymatic glycation of proteins, epigenetic modifications, senescent cells accumulation, and the enduring activation of inflammatory pathways.^20^^,^^29^, ^30^, ^31^, ^32^, ^33^, ^34^, ^35^, ^36^ Whether these phenomena explain the data presented here and whether they underly the observation that the effect of SGLT-2i against the legacy effect might be confined to the first two years after diagnosis warrants further investigation. Of note, these same mechanisms have been proposed to mediate part of the deleterious effects of hyperglycemia on the development of microvascular complications, which also suffer the legacy effect.^3^, ^4^, ^5^, ^6^, ^7^, ^8^, ^9^, ^10^ Future work is warranted to explore whether the introduction of SGLT-2i also ameliorate the legacy effect of poor glycemic control on the development of microvascular diseases, *i.e*. kidney failure, retinopathy, and neuropathy.

Despite our effort to adjust for all known risk factors, residual unmeasured confounders are inherently linked to all registry-based studies. For instance, the outcome is represented by a composite of hard outcomes with no details on the severity of each event. In addition, patients treated with SGLT-2i were younger, had a better mean renal function, and were more often on metformin and less often on sulphonylureas as background therapy, all factors that might have influenced the observed results. Also, differences in baseline disease severity are likely intertwined with the inability of reaching HbA1c targets early in the course of the disease. Another limitation might be represented by the impossibility to comment whether the results are effectively causal and the lack of a possible mechanism for such evidence. In addition, we did not perform subgroup analyses to explore eventual heterogeneity of the effect among men and women or according to age strata.^37^ Finally, risk factors levels could have changed during the follow-up phase, thus impacting the results. Thus, the results presented here should be considered as hypothesis generating and further work needs to be done to achieve more definitive answers. A prospective study with fully matched groups would help in obtaining more consistent observations. Furthermore, given the study design, we did not explore the effect of SGLT-2i in people with prevalent CVD nor in those with good glycemic control (*i.e*. with HbA1c < 7%). Of note, while the most definitive evidence for cardioprotection with SGLT-2i comes from people with established CVD,^11^, ^12^, ^13^ more work should be done to explore whether prescribing an SGLT-2i is beneficial in subjects with newly-diagnosed diabetes, good glycemic control, and free of CVD.

In summary, among patients with newly-diagnosed T2D and without CVD at baseline, we evidenced that mean HbA1c levels >7% or >8% during the 0–1, 0–2, or 0–3 years after diagnosis are associated with a greater risk of subsequent CVD compared with an HbA1c ≤ 7%. These associations are no longer visible when patients are treated with SGLT-2i in the 0–1- and 0–2-years’ time ranges. These results suggest that the legacy effect phenomenon is still observable in contemporary cohorts and that an early introduction of SGLT-2i might be able to ameliorate or even suppress the noxious long-term consequence of early, poor glycemic control on the vasculature. If confirmed in prospective studies with fully-matched populations, these findings might suggest that SGLT-2i act as disease-modifying drugs, thus advocating a wider and earlier usage for them.

## Contributors

AC and FP: conceived the idea and wrote the manuscript; GL and AN: contributed to study design, made the statistical analysis, wrote, and discussed the manuscript; RLG and CF: contributed to data analysis and reviewed the manuscript; SDC, PDB, GDC, PF, CG, RP, GR, FV: collected data, verified the underlying data, and reviewed the manuscript for intellectual content. All authors approve the final version of the manuscript.

## Data sharing statement

The datasets generated during or analyzed during the current study are available from the corresponding author on reasonable request.

## Declaration of interests

AC is on the advisory board and does consultancy and lectures for AstraZeneca, BERLIN-CHEMIE, Eli Lilly, Novo Nordisk, Mitsubishi, Roche Diagnostics, and Theras Lifetech. FP is a lecturer for BERLIN-CHEMIE. AN has received honoraria from AstraZeneca, Eli Lilly, Novo Nordisk, and research support from Alfasigma, Novo Nordisk, Sanofi, Shionogi, SOBI. GR is on the advisory board and does consultancy and lectures for Novo Nordisk, Astra Zeneca, Sanofi, Boehringer, Lilly, Mundipharma, and Sanchio. SDC received honoraria for lectures from Eli Lilly, Boehringer, Astra-Zeneca, MundiPharma, MSD, Sanofi, Novo-Nordisk, Daiichi Sankyo, and Bayer. RP received honoraria for lectures from Lilly, Boehringer, AstraZeneca, Novartis, Menarini, MSD, Sanofi, Novo-Nordisk, Vifor, Alfa-Sigma, and Bayer. P.F. reports receiving personal fees from Astra Zeneca, Lilly, Boehringer Ingelheim, Bayer, and Novo Nordisk. The remaining authors declare no conflict of interests.
