# Supplementary material for: The legacy effect of hyperglycemia and early use of SGLT-2 inhibitors: a cohort study with newly-diagnosed people with type 2 diabetes
Source: Lancet Reg Health Eur. 2023 Jun 12;31:100666. doi: 10.1016/j.lanepe.2023.100666 (PMC10398589; doi:10.1016/j.lanepe.2023.100666)
Supplement: Supplementary Tables S1–S3 [file mmc1.docx]

**Supplementary Table 1A. Characteristics of the study population by HbA1c mean (exposure period of 1 year).**

| **Characteristic** | **N** | **Overall** | **<=7** | **7.1-8.0** | **>8.0** | **p-value** |
| --- | --- | --- | --- | --- | --- | --- |
| No. of patients |  | 251339 | 150755 | 58627 | 41957 |  |
| Age at basaline (year) | 248761 | 63.6±12.4 | 63.8±12.1 | 64.2±12.7 | 61.9±13.3 | <0.0001 |
| Gender (% males) | 251338 | 143166 (57.0) | 85651 (56.8) | 33202 (56.6) | 24313 (57.9) | <0.0001 |
| HbA1c at baseline (%) | 251339 | 6.8±1.2 | 6.2±0.5 | 7.1±0.7 | 8.2±1.8 | <0.0001 |
| HbA1c at diagnosis (%) 3.0-6.9 | 251339 | 46537 (18.5) | 41606 (27.6) | 3957 (6.7) | 974 (2.3) | <0.0001 |
| HbA1c at diagnosis (%) 7.0-8.0 | 251339 | 35264 (14.0) | 22155 (14.7) | 10563 (18.0) | 2546 (6.1) |  |
| HbA1c at diagnosis (%) 8.1-9.0 | 251339 | 16050 (6.4) | 8123 (5.4) | 5076 (8.7) | 2851 (6.8) |  |
| HbA1c at diagnosis (%) >9.0 | 251339 | 41409 (16.5) | 19421 (12.9) | 11406 (19.5) | 10582 (25.2) |  |
| HbA1c at diagnosis (%) NA | 251339 | 112079 (44.6) | 59450 (39.4) | 27625 (47.1) | 25004 (59.6) |  |
| HbA1c at diagnosis (%) | 139260 | 8.4±2.3 | 7.8±2.1 | 8.9±2.2 | 10.2±2.4 | <0.0001 |
| Composite CV Outcome | 251339 | 13822 (5.5) | 7874 (5.2) | 3538 (6.0) | 2410 (5.7) | <0.0001 |
| Exposure time (year) | 251339 | 0.7±0.3 | 0.7±0.2 | 0.8±0.2 | 0.7±0.3 | <0.0001 |
| Follow-up (years) | 251339 | 4.6±2.9 | 4.6±2.9 | 4.6±2.9 | 4.4±2.9 | <0.0001 |
| BMI | 223008 | 29.8±5.6 | 29.7±5.5 | 30.0±5.7 | 30.0±6.0 | <0.0001 |
| Total cholesterol (mmol/l) | 225678 | 187.5±40.7 | 187.0±39.7 | 186.7±41.0 | 190.3±43.7 | <0.0001 |
| HDL cholesterol (mmol/l) | 219697 | 49.0±13.1 | 49.9±13.2 | 48.3±12.8 | 47.1±12.9 | <0.0001 |
| LDL cholesterol (mmol/l) | 217157 | 110.7±35.0 | 110.6±34.5 | 109.9±35.2 | 112.1±36.7 | <0.0001 |
| Triglycerides (mmol/l) | 222871 | 143.8±89.5 | 136.5±79.6 | 148.6±91.7 | 163.1±113.3 | <0.0001 |
| Diastolic blood pressure (mmHg) | 212625 | 78.9±9.9 | 78.6±9.8 | 79.0±10.0 | 79.4±10.3 | <0.0001 |
| Systolic blood pressure (mmHg) | 212726 | 133.9±18.1 | 133.5±17.8 | 134.7±18.3 | 134.3±18.8 | <0.0001 |
| Smoking | 159454 | 32274 (20.2) | 17748 (18.5) | 7972 (21.6) | 6554 (24.5) | <0.0001 |
| Microalbuminuria | 158381 | 49845 (31.5) | 27142 (29.0) | 12622 (33.3) | 10081 (37.7) | <0.0001 |
| eGFR (ml/min/1.73m^2^) < 60 | 215692 | 38344 (17.8) | 21997 (17.2) | 9770 (19.2) | 6577 (17.9) | <0.0001 |
| Antihypertensive drugs | 251339 | 119842 (47.7) | 73380 (48.7) | 28139 (48.0) | 18323 (43.7) | <0.0001 |
| Statins | 251339 | 86668 (34.5) | 52599 (34.9) | 20864 (35.6) | 13205 (31.5) | <0.0001 |

**Supplementary Table 1B. Characteristics of the study population by HbA1c mean (exposure period of 2 years).**

| **Characteristic** | **N** | **Overall** | **<=7** | **7.1-8.0** | **>8.0** | **p-value** |
| --- | --- | --- | --- | --- | --- | --- |
| No. of patients |  | 237635 | 147125 | 58310 | 32200 |  |
| Age at basaline (year) | 235468 | 64.2±12.3 | 64.5±11.9 | 64.8±12.5 | 62.3±13.5 | <0.0001 |
| Gender (% males) | 237634 | 134502 (56.6) | 83069 (56.5) | 32788 (56.2) | 18645 (57.9) | <0.0001 |
| HbA1c at baseline (%) | 237635 | 6.8±1.1 | 6.3±0.5 | 7.1±0.7 | 8.4±1.7 | <0.0001 |
| HbA1c at diagnosis (%) 3.0-6.9 | 237635 | 47311 (19.9) | 41172 (28.0) | 4962 (8.5) | 1177 (3.7) | <0.0001 |
| HbA1c at diagnosis (%) 7.0-8.0 | 237635 | 33394 (14.1) | 20233 (13.8) | 10481 (18.0) | 2680 (8.3) |  |
| HbA1c at diagnosis (%) 8.1-9.0 | 237635 | 14494 (6.1) | 7126 (4.8) | 4856 (8.3) | 2512 (7.8) |  |
| HbA1c at diagnosis (%) >9.0 | 237635 | 36104 (15.2) | 17229 (11.7) | 10613 (18.2) | 8262 (25.7) |  |
| HbA1c at diagnosis (%) NA | 237635 | 106332 (44.7) | 61365 (41.7) | 27398 (47.0) | 17569 (54.6) |  |
| HbA1c at diagnosis (%) | 131303 | 8.3±2.3 | 7.8±2.1 | 8.8±2.2 | 9.9±2.4 | <0.0001 |
| Composite CV Outcome | 237635 | 12898 (5.4) | 7408 (5.0) | 3543 (6.1) | 1947 (6.0) | <0.0001 |
| Exposure time (year) | 237635 | 1.6±0.5 | 1.6±0.5 | 1.6±0.5 | 1.5±0.5 | <0.0001 |
| Follow-up (years) | 237635 | 4.5±2.7 | 4.5±2.7 | 4.4±2.7 | 4.4±2.7 | <0.0001 |
| BMI | 216869 | 29.9±5.6 | 29.7±5.4 | 30.1±5.7 | 30.5±6.1 | <0.0001 |
| Total cholesterol (mmol/l) | 221386 | 184.5±39.5 | 184.0±38.5 | 183.7±39.7 | 188.3±43.3 | <0.0001 |
| HDL cholesterol (mmol/l) | 217475 | 49.5±13.3 | 50.4±13.4 | 48.5±13.1 | 46.8±13.1 | <0.0001 |
| LDL cholesterol (mmol/l) | 216593 | 107.4±34.0 | 107.3±33.4 | 106.5±34.3 | 109.5±36.3 | <0.0001 |
| Triglycerides (mmol/l) | 219334 | 143.0±87.6 | 135.6±78.4 | 148.6±88.7 | 167.4±116.4 | <0.0001 |
| Diastolic blood pressure (mmHg) | 209997 | 78.8±9.9 | 78.6±9.7 | 79.1±10.0 | 79.6±10.4 | <0.0001 |
| Systolic blood pressure (mmHg) | 210078 | 134.1±17.9 | 133.7±17.6 | 134.9±18.1 | 134.9±18.8 | <0.0001 |
| Smoking | 156291 | 31212 (20.0) | 17767 (18.2) | 8249 (21.6) | 5196 (25.2) | <0.0001 |
| Microalbuminuria | 171699 | 61098 (35.6) | 34889 (33.0) | 16369 (37.9) | 9840 (43.4) | <0.0001 |
| eGFR (ml/min/1.73m^2^) < 60 | 212275 | 42102 (19.8) | 24983 (19.1) | 11239 (21.4) | 5880 (20.5) | <0.0001 |
| Antihypertensive drugs | 237635 | 123446 (51.9) | 78100 (53.1) | 30423 (52.2) | 14923 (46.3) | <0.0001 |
| Statins | 237635 | 96823 (40.7) | 60785 (41.3) | 24409 (41.9) | 11629 (36.1) | <0.0001 |

**Supplementary Table 1C. Characteristics of the study population by HbA1c mean (exposure period of 3 years).**

| **Characteristic** | **N** | **Overall** | **<=7** | **7.1-8.0** | **>8.0** | **p-value** |
| --- | --- | --- | --- | --- | --- | --- |
| No. of patients |  | 210385 | 130022 | 53934 | 26429 |  |
| Age at basaline (year) | 208604 | 64.8±12.2 | 65.1±11.8 | 65.3±12.3 | 62.5±13.4 | <0.0001 |
| Gender (% males) | 210384 | 118713 (56.4) | 73056 (56.2) | 30343 (56.3) | 15314 (57.9) | <0.0001 |
| HbA1c at baseline (%) | 210385 | 6.8±1.1 | 6.3±0.6 | 7.2±0.8 | 8.5±1.7 | <0.0001 |
| HbA1c at diagnosis (%) 3.0-6.9 | 210385 | 42984 (20.4) | 36506 (28.1) | 5272 (9.8) | 1206 (4.6) | <0.0001 |
| HbA1c at diagnosis (%) 7.0-8.0 | 210385 | 29156 (13.9) | 16982 (13.1) | 9747 (18.1) | 2427 (9.2) |  |
| HbA1c at diagnosis (%) 8.1-9.0 | 210385 | 12556 (6.0) | 5924 (4.6) | 4352 (8.1) | 2280 (8.6) |  |
| HbA1c at diagnosis (%) >9.0 | 210385 | 30440 (14.5) | 14025 (10.8) | 9460 (17.5) | 6955 (26.3) |  |
| HbA1c at diagnosis (%) NA | 210385 | 95249 (45.3) | 56585 (43.5) | 25103 (46.5) | 13561 (51.3) |  |
| HbA1c at diagnosis (%) | 115136 | 8.2±2.2 | 7.7±2.0 | 8.7±2.2 | 9.7±2.4 | <0.0001 |
| Composite CV Outcome | 210385 | 11146 (5.3) | 6273 (4.8) | 3249 (6.0) | 1624 (6.1) | <0.0001 |
| Exposure time (year) | 210385 | 2.5±0.7 | 2.4±0.7 | 2.5±0.6 | 2.4±0.8 | <0.0001 |
| Follow-up (years) | 210385 | 4.3±2.5 | 4.3±2.4 | 4.2±2.4 | 4.3±2.5 | <0.0001 |
| BMI | 195109 | 30.0±5.6 | 29.7±5.4 | 30.2±5.7 | 30.7±6.1 | <0.0001 |
| Total cholesterol (mmol/l) | 199322 | 182.4±39.0 | 182.0±37.9 | 181.0±39.2 | 187.0±43.1 | <0.0001 |
| HDL cholesterol (mmol/l) | 196691 | 49.5±13.4 | 50.5±13.4 | 48.3±13.0 | 46.6±13.1 | <0.0001 |
| LDL cholesterol (mmol/l) | 196552 | 105.3±33.7 | 105.3±33.0 | 103.9±33.8 | 108.2±36.5 | <0.0001 |
| Triglycerides (mmol/l) | 197697 | 143.1±86.8 | 135.3±76.7 | 149.4±89.5 | 169.1±117.1 | <0.0001 |
| Diastolic blood pressure (mmHg) | 190188 | 78.8±9.7 | 78.5±9.5 | 79.0±9.8 | 79.7±10.3 | <0.0001 |
| Systolic blood pressure (mmHg) | 190233 | 134.4±17.8 | 133.8±17.5 | 135.2±18.1 | 135.2±18.7 | <0.0001 |
| Smoking | 141277 | 28035 (19.8) | 15935 (18.1) | 7749 (21.5) | 4351 (25.5) | <0.0001 |
| Microalbuminuria | 163240 | 64371 (39.4) | 37021 (36.7) | 17880 (41.8) | 9470 (48.0) | <0.0001 |
| eGFR (ml/min/1.73m^2^) < 60 | 192458 | 41662 (21.6) | 24774 (20.8) | 11526 (23.1) | 5362 (22.5) | <0.0001 |
| Antihypertensive drugs | 210385 | 115702 (55.0) | 72844 (56.0) | 29959 (55.5) | 12899 (48.8) | <0.0001 |
| Statins | 210385 | 94903 (45.1) | 59195 (45.5) | 25273 (46.9) | 10435 (39.5) | <0.0001 |

**Supplementary Table 1D. Characteristics of the study population by HbA1c mean (exposure period of 4 years), overall and stratified for SGLT-2i use.**

|  | **N** | **Overall** | **<=7** | **7.1-8.0** | **>8.0** | **p-value** | **SGLT2i Not user** | **SGLT2i User** | **p-value** |
| --- | --- | --- | --- | --- | --- | --- | --- | --- | --- |
| No. of patients |  | 182107 | 111318 | 48375 | 22414 |  | 174214 | 7893 |  |
| Age at baseline (year) | 180655 | 65.4±12.1 | 65.8±11.6 | 65.8±12.2 | 62.7±13.3 | <0.0001 | 65.7±12.1 | 59.1±9.9 | <0.0001 |
| Gender (% males) | 182106 | 102496 (56.3) | 62329 (56.0) | 27234 (56.3) | 12933 (57.7) | <0.0001 | 97676 (56.1) | 4820 (61.1) | <0.0001 |
| Microalbuminuria | 147787 | 63059 (42.7) | 35948 (39.8) | 18249 (45.4) | 8862 (51.2) | <0.0001 | 59660 (42.4) | 3399 (48.5) | <0.0001 |
| Antihypertensive medication | 182107 | 104994 (57.7) | 65254 (58.6) | 28294 (58.5) | 11446 (51.1) | <0.0001 | 99976 (57.4) | 5018 (63.6) | <0.0001 |
| BMI | 170891 | 30.0±5.6 | 29.7±5.4 | 30.2±5.7 | 30.9±6.2 | <0.0001 | 29.9±5.5 | 32.3±6.2 | <0.0001 |
| Total cholesterol (mmol/l) | 174280 | 180.5±38.7 | 180.2±37.6 | 178.9±38.7 | 185.6±42.9 |  | 180.7±38.6 | 177.0±39.8 |  |
| eGFR (ml/min/1.73m2) < 60 | 169425 | 39655 (23.4) | 23519 (22.7) | 11233 (24.8) | 4903 (23.9) | <0.0001 | 38654 (23.9) | 1001 (12.9) | <0.0001 |
| Follow-up (years) | 182107 | 4.0±2.3 | 4.0±2.3 | 3.9±2.2 | 4.0±2.3 | <0.0001 | 4.1±2.3 | 2.0±1.3 | <0.0001 |
| HbA1c at baseline (%) | 182107 | 6.9±1.1 | 6.3±0.6 | 7.3±0.8 | 8.6±1.7 | <0.0001 | 6.8±1.1 | 7.4±1.3 | <0.0001 |
| HbA1c at diagnosis (%) 3.0-6.9 | 182107 | 37693 (20.7) | 31282 (28.1) | 5250 (10.9) | 1161 (5.2) | <0.0001 | 36905 (21.2) | 788 (10.0) | <0.0001 |
| HbA1c at diagnosis (%) 7.0-8.0 | 182107 | 25072 (13.8) | 14093 (12.7) | 8716 (18.0) | 2263 (10.1) |  | 24184 (13.9) | 888 (11.3) |  |
| HbA1c at diagnosis (%) 8.1-9.0 | 182107 | 10598 (5.8) | 4817 (4.3) | 3801 (7.9) | 1980 (8.8) |  | 9982 (5.7) | 616 (7.8) |  |
| HbA1c at diagnosis (%) >9.0 | 182107 | 25325 (13.9) | 11213 (10.1) | 8222 (17.0) | 5890 (26.3) |  | 23168 (13.3) | 2157 (27.3) |  |
| HbA1c at diagnosis (%) NA | 182107 | 83419 (45.8) | 49913 (44.8) | 22386 (46.3) | 11120 (49.6) |  | 79975 (45.9) | 3444 (43.6) |  |
| HbA1c at diagnosis (%) | 98688 | 8.1±2.2 | 7.6±2.0 | 8.7±2.2 | 9.6±2.4 | <0.0001 | 8.1±2.2 | 9.3±2.4 | <0.0001 |
| HDL cholesterol (mmol/l) | 172432 | 49.5±13.4 | 50.6±13.4 | 48.3±13.1 | 46.4±13.1 | <0.0001 | 49.6±13.4 | 46.9±12.1 | <0.0001 |
| LDL cholesterol (mmol/l) | 172697 | 103.6±33.3 | 103.6±32.7 | 101.9±33.4 | 106.9±36.0 | <0.0001 | 103.8±33.3 | 99.0±33.4 | <0.0001 |
| HbA1c mean in exposure period ≤7.0 | 182107 | 111318 (61.1) |  |  |  |  | 108757 (62.4) | 2561 (32.4) | <0.0001 |
| HbA1c mean in exposure period 7.1-8.0 | 182107 | 48375 (26.6) |  |  |  |  | 45093 (25.9) | 3282 (41.6) |  |
| HbA1c mean in exposure period >8.0 | 182107 | 22414 (12.3) |  |  |  |  | 20364 (11.7) | 2050 (26.0) |  |
| Diastolic blood pressure (mmHg) | 167301 | 78.6±9.6 | 78.3±9.5 | 78.8±9.7 | 79.6±10.2 | <0.0001 | 78.6±9.6 | 79.9±9.9 | <0.0001 |
| Systolic blood pressure (mmHg) | 167330 | 134.4±17.7 | 133.9±17.4 | 135.1±17.9 | 135.4±18.7 | <0.0001 | 134.4±17.7 | 133.8±17.8 | <0.0001 |
| Smoking | 124032 | 24527 (19.8) | 13694 (17.9) | 7010 (21.4) | 3823 (26.1) | <0.0001 | 23032 (19.5) | 1495 (25.5) | <0.0001 |
| Composite CV Outcome | 182107 | 9242 (5.1) | 5130 (4.6) | 2785 (5.8) | 1327 (5.9) | <0.0001 | 8958 (5.1) | 284 (3.6) | <0.0001 |
| Exposure time (year) | 182107 | 3.4±0.9 | 3.3±0.9 | 3.5±0.8 | 3.3±1.0 | <0.0001 | 3.4±0.9 | 3.7±0.4 | <0.0001 |
| Triglycerides (mmol/l) | 173064 | 142.6±85.7 | 134.4±74.6 | 148.7±89.2 | 169.8±117.3 | <0.0001 | 141.5±84.5 | 165.7±105.1 | <0.0001 |
| DPP4i | 182107 | 25375 (13.9) | 9325 (8.4) | 11459 (23.7) | 4591 (20.5) | <0.0001 | 23890 (13.7) | 1485 (18.8) | <0.0001 |
| Glinides | 182107 | 8928 (4.9) | 3729 (3.3) | 3309 (6.8) | 1890 (8.4) | <0.0001 | 8723 (5.0) | 205 (2.6) | <0.0001 |
| GLP1-RAs | 182107 | 7652 (4.2) | 2379 (2.1) | 3296 (6.8) | 1977 (8.8) | <0.0001 | 6413 (3.7) | 1239 (15.7) | <0.0001 |
| Acarbose | 182107 | 4846 (2.7) | 2177 (2.0) | 1815 (3.8) | 854 (3.8) | <0.0001 | 4637 (2.7) | 209 (2.6) | 0.9408 |
| Insulin | 182107 | 35168 (19.3) | 11483 (10.3) | 11984 (24.8) | 11701 (52.2) | <0.0001 | 32186 (18.5) | 2982 (37.8) | <0.0001 |
| Metformin | 182107 | 123181 (67.6) | 68641 (61.7) | 37809 (78.2) | 16731 (74.6) | <0.0001 | 115691 (66.4) | 7490 (94.9) | <0.0001 |
| Sulphonylureas | 182107 | 27352 (15.0) | 9270 (8.3) | 10807 (22.3) | 7275 (32.5) | <0.0001 | 25888 (14.9) | 1464 (18.5) | <0.0001 |
| Statins | 182107 | 88618 (48.7) | 54493 (49.0) | 24658 (51.0) | 9467 (42.2) | <0.0001 | 83712 (48.1) | 4906 (62.2) | <0.0001 |

**Supplementary Table 2. Lack of interaction between use or non-use of SGLT-2i in the 0-4 years exposure period.** Adjusted hazard ratios (HR) with the relative 95% confidence interval (CI) and the p value, derived from the Cox regression analyses exploring the associations between glycemic control and the risk of the CVD at follow-up in the overall cohort and in patients stratified according to use of SGLT-2i during the exposure phase or not users, in the 0-4 years exposure period, along with the relative p for interaction. HbA1c ≤ 7% is the reference.

| **Exposure 0-4 years** |  | |  |
| --- | --- | --- | --- |
| **Overall cohort** | **HR (95% CI)** | **p-value** |  |
| HbA1c (%) 7.1-8.0 | 1.17 (1.11-1.23) | <.0001 |  |
| HbA1c (%) >8.0 | 1.29 (1.21-1.38) | <.0001 |  |
| **SGLT2i Users** | **HR (95% CI)** | **p-value** | **P for interaction** |
| HbA1c (%) 7.1-8.0 | 0.84 (0.64-1.11) | 0.22 | 0.14 |
| HbA1c (%) >8.0 | 1.13 (0.83-1.52) | 0.44 |  |
| **SGLT2i Not users** | **HR (95% CI)** | **p-value** |  |
| HbA1c (%) 7.1-8.0 | 1.18 (1.12-1.24) | <.0001 |  |
| HbA1c (%) >8.0 | 1.30 (1.22-1.40) | <.0001 |  |

**Supplementary Table 3. Discontinuation rate of SGLT-2i prescription at the end of the follow-up in the 4 exposure periods considered.**

|  | **SGLT2i discontinuation rate, number (%)** |
| --- | --- |
| **1 year exposure** | 25532 (12.6) |
| **2 years exposure** | 24485 (13.1) |
| **3 years exposure** | 21854 (13.5) |
| **4 years exposure** | 18752 (13.7) |
